# PENILE PAPULONECROTIC TUBERCULID: REVISITED

**DOI:** 10.4103/0019-5154.44790

**Published:** 2008

**Authors:** Amiya Kumar Nath, Sakthi Kandan Janakiraman, Abhijit Chougule, Devinder Mohan Thappa

**Affiliations:** *From the Department of Dermatology and STD, Jawaharlal Institute of Postgraduate Medical Education and Research (JIPMER), Pondicherry - 605 006, India*

Sir,

Papulonecrotic tuberculid causing penile ulcers is extremely rare.^1^ Herewith, we report a case of papulonecrotic tuberculide of the penis in a 56-years-old male.

A 56-year-old married male patient was referred to our department with multiple asymptomatic non-healing ulcers over the glans penis of one month duration. He was a heterosexual individual and his wife did not have any genital lesions or discharge. The patient denied any history of pre-marital and extra-marital sexual contact.

**Fig. 1:**
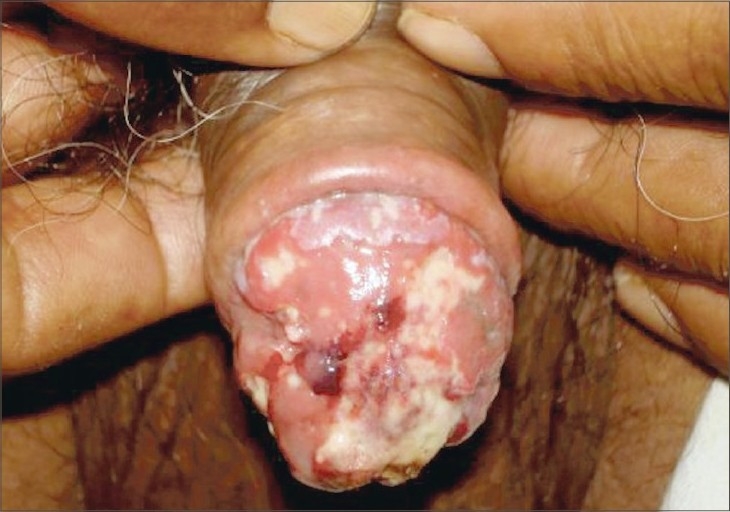
Glans penis showing multiple ulcers

On physical examination, there were multiple, superficial and deep tender ulcers on the glans penis with ragged, irregular margins and floor covered with necrotic yellow slough (Fig. 1). The urethral meatus was hidden by these ulcerative lesions. Rest of the genital examination was normal. There was no inguinal lymphadenopathy. His systemic examination was unremarkable.

The hemogram revealed elevated erythrocyte sedimentation rate (50 mm in the first hour). Tuberculin (Mantoux) test was strongly positive (20 mm × 20 mm). Gram stain of the discharge from the ulcers demonstrated pus cells, Gram positive cocci, and Gram negative bacilli and discharge from the ulcers grew *Staphylococcus aureus*, *Escherichia coli*, and *Enterococcus faecalis*. Ziehl Neelsen stain of the pus did not demonstrate any acid-fast bacilli (AFB). Tzanck smear from ulcer was negative for multinucleated giant cells. Urine sediment examination for AFB and urine culture were noncontributory. Radiological and ultrasound evaluation of the genitourinary system was normal. HIV antibodies test and VDRL test were nonreactive. Systemic evaluation for any focus of tuberculosis was unremarkable.

**Fig. 2:**
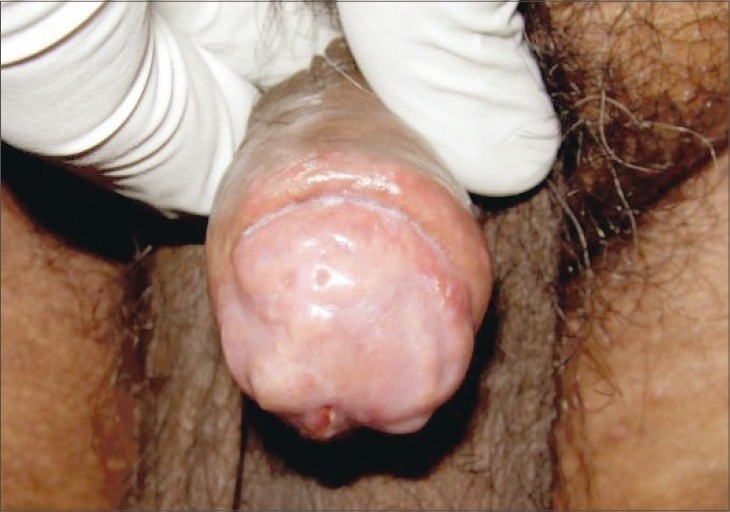
Healing with residual depressed scars after 3 months of therapy

Biopsy from the edge of the ulcer (glans penis) revealed ulcerated epidermis. In the deep dermis, by the side of ulceration, there were caseating tuberculous granulomas along with perivascular infiltrate with vessel wall thickening and endothelial cell swelling. Fite stain for AFB was negative. These features were consistent with papulonecrotic tuberculide. AFB culture of biopsy specimen was negative. Repeated courses of antibiotic therapy did not yield desired results; hence, antitubercular therapy was initiated keeping in mind the possibility of papuloneurotic tuberculide of the penis. Four-drug combination therapy of rifampicin, isoniazid, pyrazinamide, and ethambutol was given for initial 2 months followed by combination of rifampicin and isoniazid to complete total 6 months of standard antitubercular therapy. The lesions started responding to therapy in next two weeks and complete healing with residual depressed scars occurred after three months of therapy (Fig. 2).

Tuberculosis of the penis is rare, even in third world countries where the prevalence of tuberculosis remains relatively high.^1^ Till 1999, only 161 cases of penile tuberculosis were reported.^2^ Understandably, papulonecrotic tuberculide involving the glans penis is even rarer.^3^,^4^

Tuberculides are hypersensitivity reactions to *Mycobacterium tuberculosis* or its products in individuals with good immunity.^5^ These cases are characterized by positive tuberculin test, evidence of present or past tuberculosis, absence of *M. tuberculosis* in the skin lesions and response to antitubercular treatment.^5^ However, a focus of tuberculosis elsewhere in the body may not be demonstrable in majority of the cases with papulonecrotic tuberculide as in our case.^2^,^6^

Papulonecrotic tuberculides are characterized by recurrent eruptions of asymptomatic, dusky red papules, which ulcerate and crust, and heal after a few weeks with varioliform scarring.^5^,^7^ These occur symmetrically and predominantly on the extensor aspects (legs, knees, elbows, hands and feet) of the extremities. Other areas that may be rarely affected by papulonecrotic tuberculides are the ears, face, buttocks, perniotic areas and penis.^5^ In Japan, penile tuberculide has been considered a disease entity.^4^

Thus, it is important to remember tuberculosis as an underlying cause of penile ulcers, more so in countries like India where prevalence of tuberculosis is still high.
